# An Experimental and Numerical Study on the Use of Chirped FBG Sensors for Monitoring Fatigue Damage in Hybrid Composite Patch Repairs

**DOI:** 10.3390/s21041168

**Published:** 2021-02-07

**Authors:** Rodolfo L. Rito, Stephen L. Ogin, Andrew D. Crocombe

**Affiliations:** Department of Mechanical Engineering Sciences, Faculty of Engineering and Physical Sciences, University of Surrey, Guildford U2 7XH, UK; rodolfo.rito@gmail.com (R.L.R.); a.crocombe@surrey.ac.uk (A.D.C.)

**Keywords:** structural health monitoring, chirped fibre bragg grating, fibre reinforced plastic, finite element analysis (FEA), fatigue delamination

## Abstract

In this paper, chirped fibre Bragg grating (CFBG) sensors used to monitor the structural health of a composite patch used to repair an aluminium panel is presented. To introduce damage, a notch was produced at the centre of an aluminium panel. The repair consisted of bonding a pre-cured composite patch to the host panel using an aerospace-grade film adhesive; the sensor was embedded in the bond-line during fabrication of the repair. The repaired panels were subjected to tension-tension loading in fatigue. Cracks initiated and grew from both ends of the notch in the aluminium panels and the fatigue loading was stopped periodically for short periods of time to record the reflected spectra from the sensor. It was found that perturbations in the reflected spectra began to occur when the crack was within about 2 to 3 mm of the sensor location; after the crack passed the sensor location, the perturbations essentially stabilised. Predicted reflected spectra have been found to be in good agreement with the experiment, confirming that CFBG sensors can detect crack growth in patch-repaired panels.

## 1. Introduction

The service life of cracked metallic structural components can be extended using adhesively bonded repair techniques. The patch repair is a well-known technique that consists of bonding a composite patch to the substrate to produce a symmetric or asymmetric repair, i.e., a single- or double-sided patch repair, respectively. The advantages of a bonded repair over a mechanical fastened repair are that the former is lighter and more structurally efficient due to better load distribution, and the reduction of stress concentrations lead to better fatigue performance. Aluminium alloys are widely used in a wide range of industries, especially in various high-performance structures (e.g., in aerospace and automotive structures). The possibility of bonding dissimilar high-performance materials such as aluminium alloys and composites has also led to a rapidly increasing interest in bonded repair technologies.

Equally important for producing strong and durable bonded composite repairs [1,2,3,4,5,6] is the need to monitor these repairs during their lifecycle. The difficulty with performing traditional non-destructive evaluation (or testing) of bond-lines has led to the need for developing new ways to monitor the “health” of a composite repair system. In addition, the possibility of having an intelligent system consisting of real time monitoring while in service would be valuable from an operations perspective, for example, an aircraft would be inspected periodically while on the ground and in preparation for the next flight. The health-monitoring system would provide information on the state of a bonded repair, which would result in an assessment as to whether the aircraft should, or should not, be grounded due to fatigue damage. This has the potential to minimise the time the aircraft is grounded and therefore greatly reduce maintenance costs. A robust health-monitoring system would certainly be a significant step forward towards allowing bonded repairs to be more widely used. Fibre Bragg grating (FBG) sensors, which are widely used for monitoring strain in structures, have also been widely suggested as possible solutions for many damage-monitoring applications in composite materials [7,8,9,10]. Whereas strain measurements derived from reflected spectra are straightforward for FBG sensors, monitoring damage produces additional complexities, and it is often necessary to use the broadening of a reflected peak, or a change in intensity, as measurements of damage. On the other hand, chirped fibre Bragg grating (CFBG) sensors, although more expensive, are generally easier to interpret with regard to damage [11,12].

The use of embedded chirped fibre Bragg grating (CFBG) sensors for strain sensing is a technique that not only offers the in situ strain readings via a “plug-and-play” system, but also enables the possibility of having a network of sensors potentially providing real-time monitoring. Furthermore, these sensors are very thin (minimising the impact on the structural performance of the host material), do not cause any electro-magnetic interference to other systems, and the technology readiness for mass production and commercialisation is well established. The use of CFBG sensors offers the possibility of not only indicating when fatigue-induced delamination has initiated, but also of predicting delamination growth.

The first time a CFBG sensor was used to monitor damage initiation and propagation was when Okabe et al. [13] embedded a sensor in carbon fibre reinforced polymer (CFRP) cross-ply laminates. It was found that this technique enabled both the identification of cracks and the determination of the location of crack development (damage accumulation) in relation to the length of the sensor. Palaniappan et al. [11] performed a similar study but using glass fibre reinforced polymer (GFRP) cross-ply laminates showing the capability of the technique to detect the initiation and growth of delaminations. Embedded CFBG sensors have also been used in single-lap joints to detect delamination and manufacturing defects in the form of cracks or voids [12,14,15]. For example, Palaniappan et al. [14] embedded a CFBG sensor in one of the adherends of a GFRP lap-joint; the results showed good agreement between the dip in the spectrum and the actual position of a delamination front growing under fatigue loading. More recently and for the first time, Rito et al. [16] tested the robustness of the technique by embedding a CFBG sensor to monitor GFRP-to-GFRP patch repairs, which showed more complex failure mechanisms and hence a more complex strain field. The results showed that progression of the fatigue damage was easily seen within the reflected spectra at the low-wavelength end, and predictions of the strain distributions enabled the reflected spectra obtained during damage growth to be understood, with good agreement between theory and experiment.

In the work presented here, an embedded CFBG sensor was used for monitoring the fatigue damage in a bonded composite repair that consists of a composite patch bonded to an aero-grade aluminium alloy panel. This technique involved embedding a CFBG sensor within the repair during the repair fabrication; the reflected spectrum provides information on the “health” of the repair.

## 2. Experimental Study

### 2.1. Experimental Procedure

**Figure 1:**
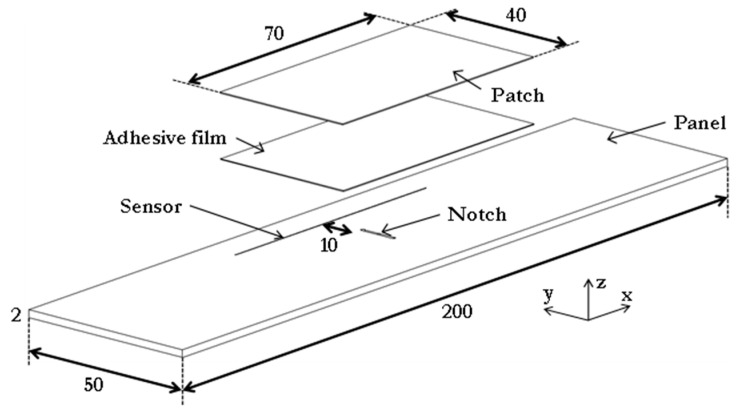
Schematic view of the repaired panel with the embedded chirped fibre Bragg grating (CFBG) sensor.

A 2 mm thick panel made of aluminium (2014-T6) was cut with a waterjet into coupons 50 mm wide and 200 mm long. In this machining operation, a notch (11 mm long and 1 mm wide) was also created at the centre of each coupon to introduce damage. The GFRP patches were obtained from a panel fabricated from YO227 E-glass 8-harness satin fabric impregnated in Shell Epikote 828 epoxy resin, creating a transparent material. A single fabric layer [(0,90)] was impregnated using the hand lay-up technique and cured at 100 °C for 3 h under a pressure of 12 kPa. The result was a laminated panel 0.25 mm thick that was cut with a waterjet into pre-cured patches of dimensions 40 mm wide and 70 mm long. Prior to the repair of the coupon, both the host material and patch were abraded and degreased thoroughly using abrasive paper and isopropyl alcohol. The 60 mm long CFBG sensors (80% reflectivity and uncoated) were supplied by Teraxion. The sensors were degreased with isopropyl alcohol, dried with Kimwipes^®^ tissue, and placed parallel to the *x* (loading) direction, 10 mm away from the notch tip, as shown in Figure 1. Each sensor had an FC/PC connector installed at the low-wavelength (*lw*) end for connecting to the optical system (i.e., the W4 FBG interrogator). Two sheets of adhesive film (FM-73 OST from Cytec) of the same dimensions as the patch were used for the repair. After assembling the adhesive and the GFRP patch (as shown by the arrows in Figure 1), the repair was cured at 120 °C for 1 h at 11 kPa following the adhesive manufacturer’s guidelines.

**Figure 2:**
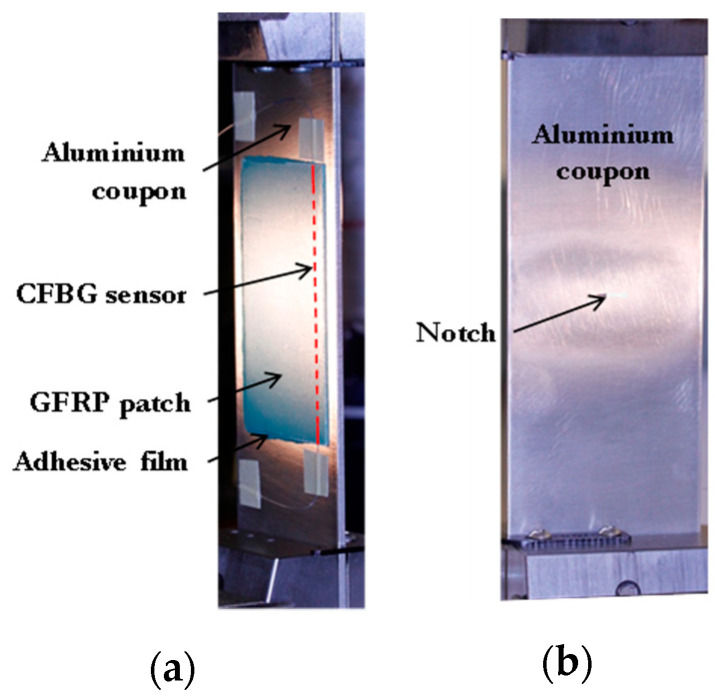
Photograph of the (**a**) patched and (**b**) unpatched faces of a repaired coupon subjected to tensile load.

To achieve crack growth from the roots of the machined notch (located under the repair), the repaired aluminium panel was fatigue loaded with tension parallel to the sensor direction (see Figure 2). The load had a sinusoidal waveform with a maximum and minimum value of 10 kN and 5 kN, respectively (R-value of 0.5), at a frequency of 10 Hz. The fatigue test was periodically stopped to record the reflected spectrum with the repaired coupon held under load, and to take photographs of the repaired region. The experimental observations and optical results are presented and discussed in the following sections.

### 2.2. Visual Damage Observations

**Figure 3:**
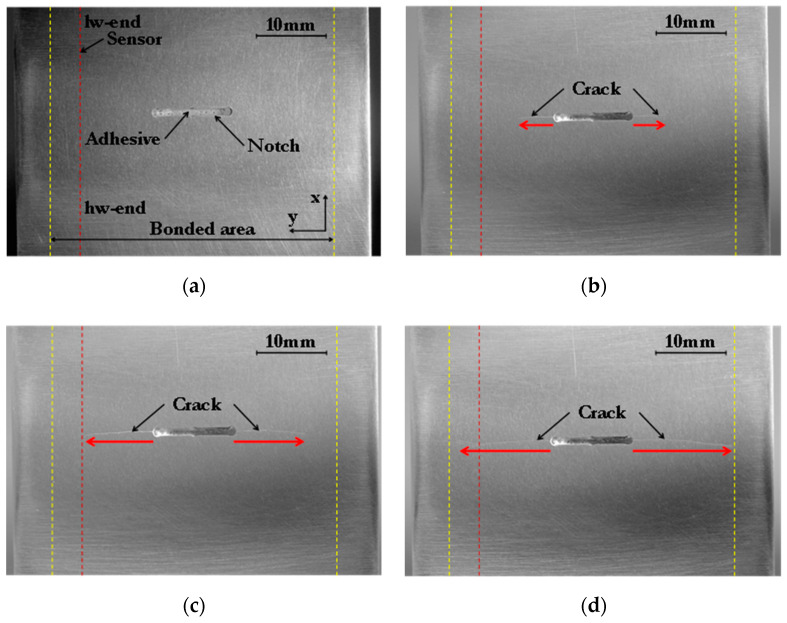
Photographs of the unpatched face of the repaired coupon after (**a**) 0, (**b**) 86,000, (**c**) 101,000, and (**d**) 106,000 fatigue cycles showing the approximate location of the sensor (red dashed line) and patch boundaries (yellow dashed lines).

During fatigue loading, damage developed in the aluminium parent panel. Figure 3 shows photographs taken on the unpatched face of a coupon for different degrees of fatigue damage. During the fatigue test, cracks initiated from the notch ends and grew approximately perpendicular to the loading direction, i.e., towards the edges of the repair, at similar rates as shown by the red arrows in Figure 3. The sensor location on the other face of the aluminium panel is shown in Figure 3 by the red dotted line, and the edges of the patch repair (also on the other face of the panel) are indicated by the yellow dashed lines. After 106,000 fatigue cycles, when the crack on the left was 12.7 mm long (Figure 3d), the crack had passed the location of the sensor, which was 10 mm from the initial notch.

**Figure 4:**
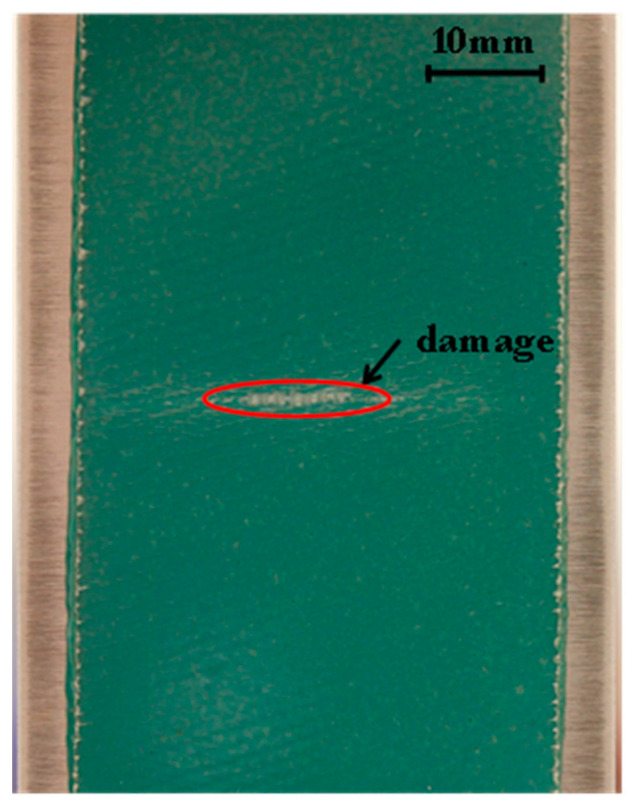
Photograph of the patched face of the repaired coupon after 106,000 fatigue cycles.

Figure 4 shows a photograph of the patched face of specimen 1 after 106,000 fatigue cycles, at which point the fatigue cracks extended almost the full width of the aluminium parent panel. It can be seen that damage occurred in the GFRP patch, especially in the notch region, far away from the sensor. This damage in the patch was clearly observable after the cracks in the aluminium panel were approximately 8 mm long. Matrix cracking and associated local delaminations in the patch were observable, especially in the notch region, but there was no evidence of fibre fracture. This is consistent with the finite element (FE) modelling (see Section 3.2) that suggests a maximum composite strain of 1.7% in the notch region, which is lower than the patch failure strain of 2% [17]. In the next section, the optical results obtained from the fatigue test are presented and explained.

### 2.3. Reflected Spectra Produced under Quasi-Static Loading

**Figure 5:**
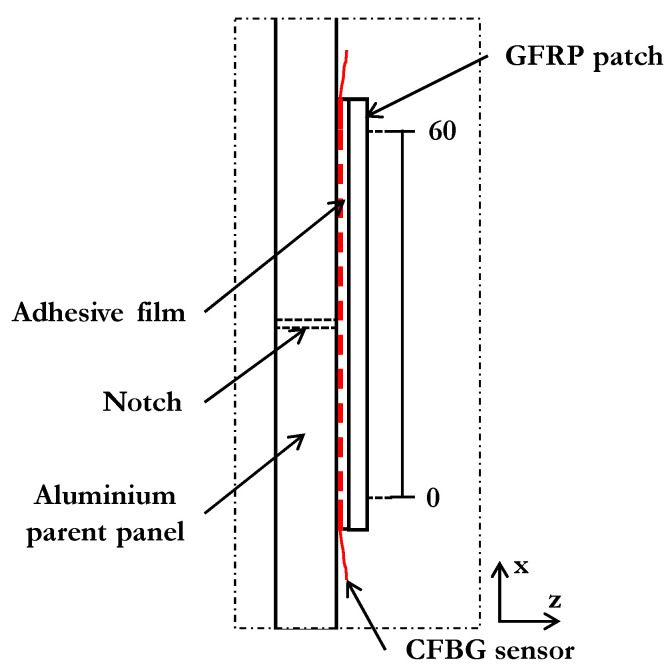
Schematic of the edge view of the specimen along the sensor line (not to scale).

As indicated earlier, the CFBG sensor extended 30 mm on each side of the notch/crack path (Figure 5 shows an edge view of the patched panel). The reflected spectra were recorded before embedding the sensor (original spectrum), and then again after embedding it, for two different loading conditions, i.e., when the specimen was (i) unloaded and (ii) loaded at 6 kN.

**Figure 6:**
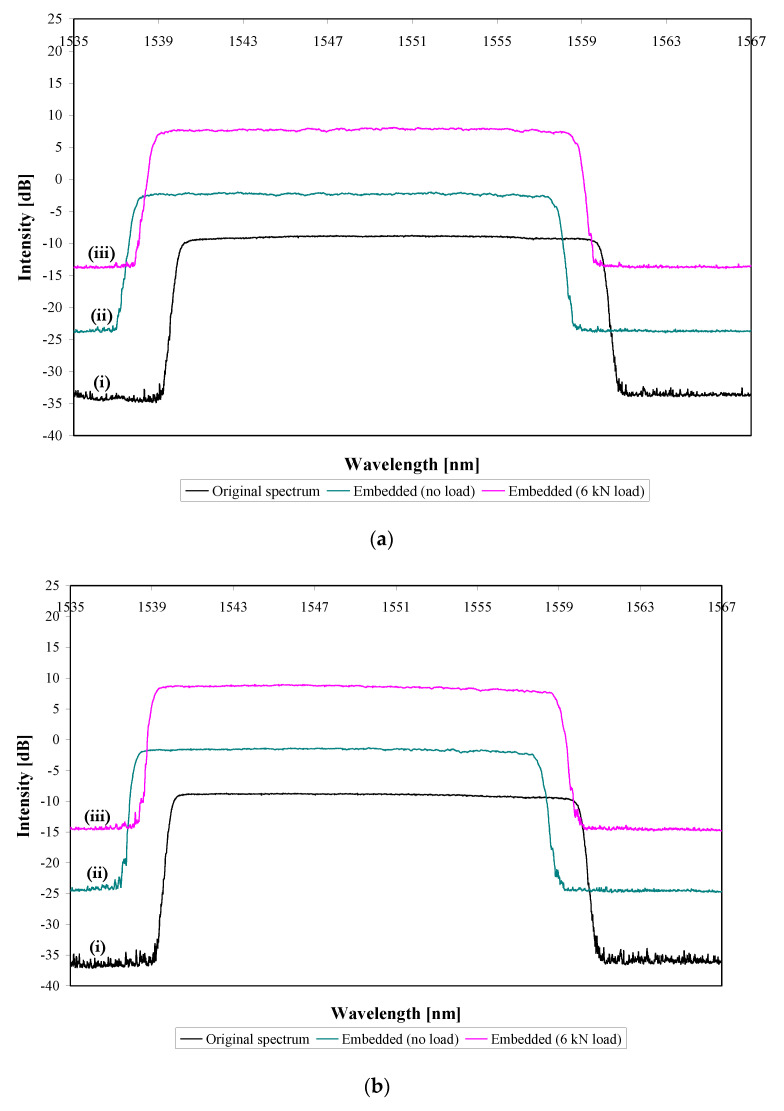
Reflected spectra of repaired but undamaged specimens (**a**) S1 and (**b**) S2 with (i) the sensor unembedded and unloaded, (ii) the sensor embedded and the specimen loaded at 0 kN, and (iii) the sensor embedded and specimen loaded at 6 kN.

Figure 6a,b shows the original spectrum (before embedding) and the spectra for no load and for a 6 kN load for specimens S1 and S2 prior to fatigue loading. The intensities of the embedded reflected spectra have been shifted by +10 and +20 dB from the original spectrum for better visualisation. In Figure 6a, the original reflected spectrum shifted about 2 nm to lower wavelengths after fabrication of the repaired specimen. This shift is related to the compressive strains formed during the cooling stage of the adhesive bonding. The coefficient of thermal expansion of the FM-73 OST adhesive film, the GFRP, and the aluminium parent laminate are all greater than that of the sensor, so that when the bonded-in sensor is cooled from 120 °C to room temperature, the surrounding materials contract more than the sensor does and hence the sensor is put into compression. The wavelength/strain ratio for CFBG sensors is about 1 × 10^−3^ nm/µε [18]. Therefore, a wavelength shift of 2 nm indicates a compressive strain of 2000 µε. The magnitude of the locked-in thermal strain can be estimated using a simple closed-form, one dimensional approach [19]. This gives 2300 µε, which is in reasonable agreement with the 2000 µε measured from the CFBG sensor. When loaded, the spectrum, of course, shifts to higher wavelengths. For example, for specimen S1 loaded to 6 kN, a shift of about 0.9 nm occurred corresponding to about 900 µε. Again, simple hand-calculations predict 830 µε, which is in reasonable agreement with the sensor measurements. Similar behaviour was seen for S2 (Figure 6b).

### 2.4. Spectra Generated during Damage Growth

**Figure 7:**
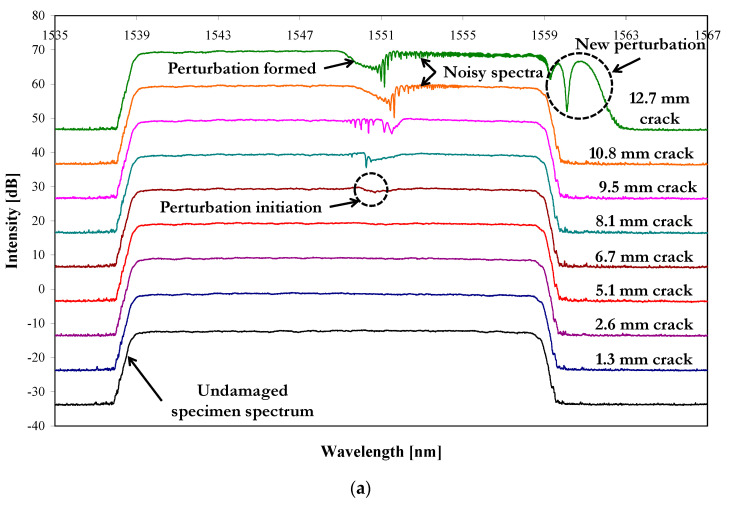

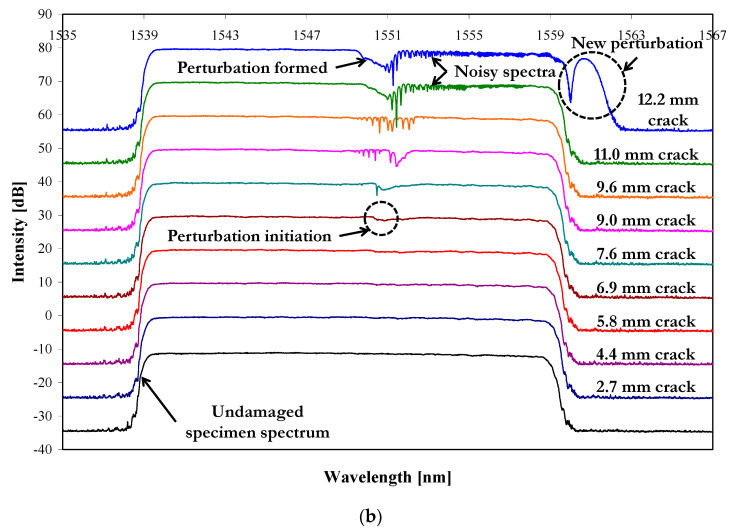
(**a**) Experimentally-recorded reflected spectra for different degrees of damage, from 1.3 mm to 12.7 mm crack length, in specimen 1 (S1) loaded at 6 kN and (**b**) experimentally recorded reflected spectra for different degrees of damage, from 2.7 mm to 12.2 mm crack length, in specimen 2 (S2) loaded at 6 kN.

The purpose of embedding the CFBG sensor into the patch repair was to investigate its ability to monitor damage initiation and growth. Figure 7a shows the reflected spectrum for specimen S1 recorded before the start of the fatigue test (undamaged specimen spectrum) and the reflected spectra recorded periodically during the fatigue test as the fatigue crack grew towards, and beyond, the location of the sensor. These spectra were taken at crack lengths of 1.3, 2.6, 5.1, 6.7, 8.1, 9.5, 10.8, and 12.7 mm. The fatigue test was periodically interrupted in order for these measurements to be made using an optical microscope and to photograph the specimen. Similar results are shown in Figure 7b for specimen S2. All the spectra were obtained with the fatigue test interrupted and the specimen loaded statically at 6 kN. In Figure 7a,b, the spectra have been shifted +10 dB on the intensity scale, starting from the undamaged spectrum, for easier visualisation. Figure 7a,b shows that, with increasing fatigue damage up to a crack length of about 7 mm, the sensor was unable to detect the fatigue crack, as shown by the uniform reflected spectra. For a crack length of about 8 mm and above, a perturbation within the reflected spectra formed, as shown by the dashed circle in Figure 7a,b. With further increase of damage, this perturbation increased in depth until it remained constant at a crack length of about 11 mm (i.e., when the crack had just passed the sensor). This indicates that when the cracks had passed the sensor, the perturbation did not undergo further changes despite the increasing stress levels at the tip of the crack (naturally the magnitude of the stress at the crack tip increased with increasing crack length for the applied 6 kN load). It is possible that the increase of the stress level at the crack tip was detected by the sensor by showing increasing noise in the spectrum at higher wavelengths and, in particular, the formation of a new perturbation beyond the high-wavelength end of the spectrum. The changes in the spectrum are predicted and discussed further in the next section.

## 3. Prediction of the Reflected Spectra for Undamaged and Damaged Patch Repairs

### 3.1. FE Model and Predicted Reflected Spectra of the Repaired Coupon without Damage

In order to be able to predict the optical results, FE modelling was performed using Abaqus^®^. The coupon was modelled using solid elements with orthotropic material properties for the GFRP patch and isotropic properties for the other components. The mechanical properties used in the FE models (produced based on the nominal dimensions of the real coupons) for the aluminium 2014-T6, the eight-harness GFRP laminate and the FM73 OST adhesive film are shown in Table 1 and Table 2, taken from [12,14,20], respectively. Due to bonding of dissimilar materials with significantly different coefficients of thermal expansion (CTE), the FE models considered the temperature drop that occurred in the cooling stage from the adhesive curing temperature at 120 °C to room temperature at 20 °C, i.e., a temperature drop of 100 °C. Table 3 shows the CTE of each material.

**Figure 8:**
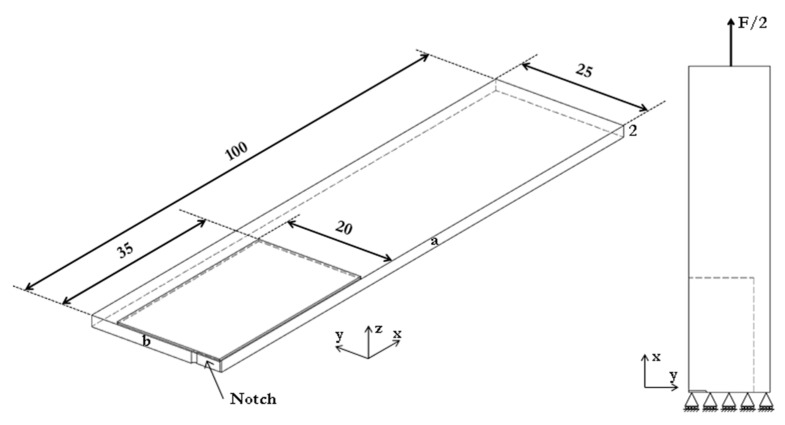
Schematic of the finite element (FE) model with no damage.

Due to symmetry, just one quarter of the patch-repaired panel was modelled with symmetric boundary conditions on faces *a* and *b* (not including the notch face), as shown in Figure 8. The model of the aluminium panel was 100 mm long, 25 mm wide, and 2 mm thick and the notch was 5.5 mm long and 0.5 mm wide. Similarly, the GFRP patch laminate and the FM-73 OST adhesive film were 35 mm long and 20 mm wide as shown in Figure 8. All the parts that comprise the system (i.e., the aluminium parent panel, the GFRP patch laminate, and the FM-73 OST adhesive film) were joined using tie constraints. The bond-line, which consisted of two layers of the FM-73 OST adhesive film, was 0.2 mm thick. The pre-cured patch (which consisted of a single ply) was 0.25 mm thick. For the modelling, a tensile load of 3 kN was applied to the undamaged and damaged models, as in the experiments (as only half the coupon width was modelled, only half the load was applied). On the face where the load was applied, a coupling constraint was used ensuring that the x-displacements of all the face nodes were the same. This was to enable the load to be applied across the entire cross-section of the model and replicate realistically the loading condition applied experimentally.

**Figure 9:**
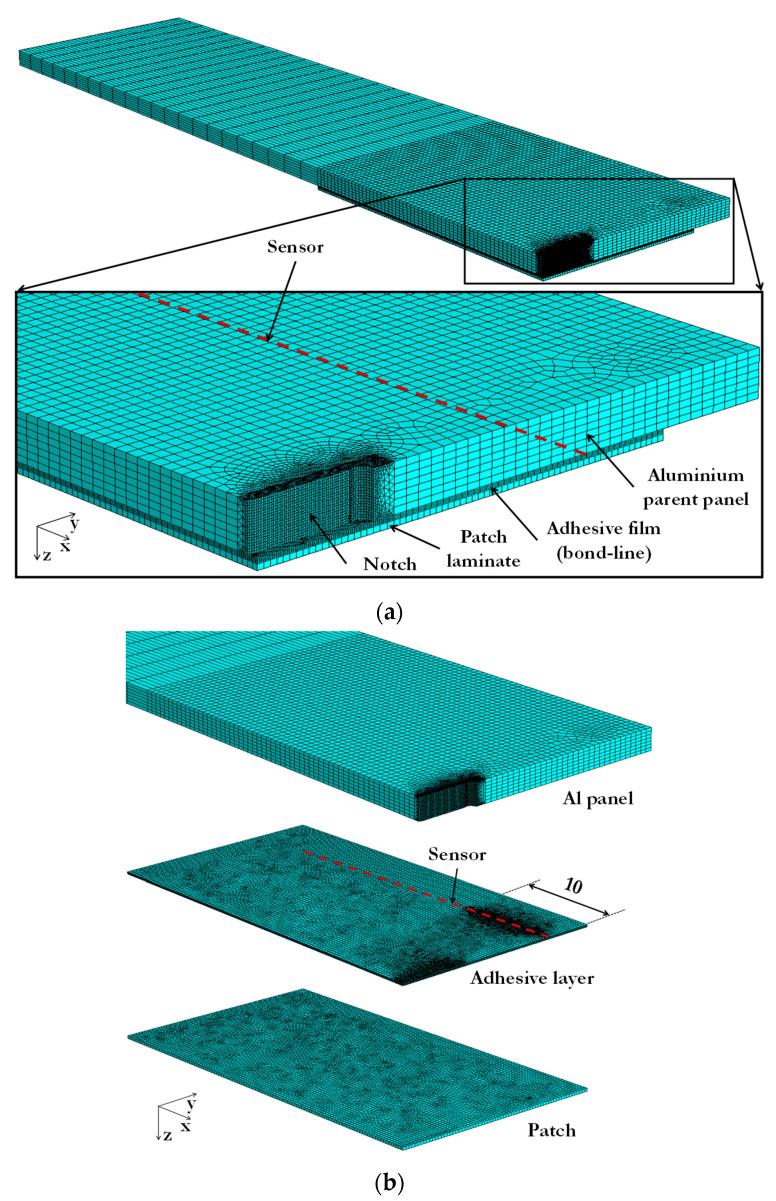
(**a**) General and detailed view of the FE mesh of the Al/GFRP patch repair model and (**b**) individual mesh of the aluminium parent panel, adhesive layer, and patch laminate.

The FE mesh of the undamaged model is shown in Figure 9a, where the position of the sensor (not modelled) is also shown. The mesh was particularly refined (with elements being 0.1 mm long) along the centre line position of the sensor (i.e., in the adhesive film adjacent to the aluminium parent panel) for better accuracy, and it was optimised throughout the remaining model for faster analysis. Due to the complex shape and high stresses expected around the notch due to tensile loading, the mesh was also refined in this region. This is especially important considering that high strains are expected at the notch and crack tips (i.e., for the undamaged and damaged models, respectively). No assessment of the effect of the mesh size on the results was undertaken because the sensor required a very refined mesh size already. The detailed view of the bond-line in Figure 9a shows this refined mesh in the repaired model. The individual mesh of each part that forms the coupon is shown in Figure 9b, i.e., the parent panel, the adhesive layer, and the patch laminate. In this figure, the fine mesh in the adhesive layer along the position of the sensor for the first 10 mm from the *yz* symmetry plane and in the notch region can be observed. The patch had a uniform mesh across the entire part. In most of the model, hexahedral elements were used; however, tetrahedral elements were used around the notch in the parent panel to obtain a better mesh transition between the local fine mesh and more remote coarse mesh, and due to the complex shape of the notch. Quadratic (2nd order) elements were used across the entire mesh as these are more accurate than linear (1st order) elements.

**Figure 10:**
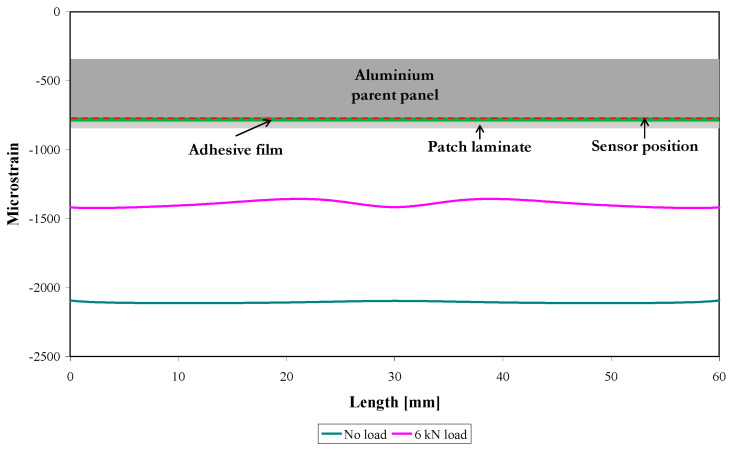
Predicted strain profiles along the position of the 60 mm sensor of the undamaged model when loaded at 0 kN and 6 kN.

The strain profiles along the sensor location, derived from the FE modelling, for the undamaged patch repair loaded at 0 kN (no load) and 6 kN are shown in Figure 10, with an inset at the top of the figure indicating the bonded region schematically. Note that the strain from the symmetric “quarter” FE model has been mirrored about the coupon centre to provide data along the entire sensor. For this situation, the sensor is located about 10 mm from the notch tip, so the expected strain profile is essentially the far-field strain in the patched panel, distant from the notch. The unloaded profile shows a uniform strain of approximately −2100 µε (−0.2%), which is the residual compressive strain caused by the temperature drop of 100 °C (to model the cooling stage of the adhesive from 120 °C to 20 °C). This is consistent with the hand-calculated value (2307 µε) discussed in Section 2.3. The loaded strain profile in Figure 10 also shows a uniform strain, this time of approximately of −1420 µε. The compressive residual strain has reduced by 680 µε. A simple estimate of the strain, considering a uniform aluminium panel (i.e., no repair and notch) of the same dimensions, subjected to a tensile load of 6 kN, gives a strain of about 830 µε, which is in reasonable agreement with the 680 µε obtained from the FE modelling; it is probable that the repair and notch account for the difference. The small undulation seen in the 6 kN loaded strain profile in Figure 10 between 10 and 50 mm of the sensor is a consequence of the strain field change due to the notch.

The longitudinal strain profiles obtained from the FE modelling were transformed into grating spacing and position data, using the Bragg equation (Equations (1) and (2) (taken from [24]) where *λ_B_* is the Bragg wavelength, *n_eff_* is the effective refractive index, *Λ* is the grating spacing, *ε* is the uniaxial strain, *P_e_* is the photo-elastic coefficient, *n_eff,_*_0_ is the unstrained effective refractive index, and ∆*n_eff_* is the change in the effective refractive index. The photo-elastic coefficient, *P_e_*, was determined from Equation (3) (taken from [25]) where *P*_11_, *P*_12_, and *ν* are shown in Table 4 [13].

(1) $$\lambda_{B}=2n_{eff}\Lambda$$

(2) $$\Delta n_{eff}=n_{eff,0}p_{e}\varepsilon$$

(3) $$p_{e}=\left(\frac{n_{eff,0}^{2}}{2}\right)\left[P_{12}-\upsilon \left(P_{11}+P_{12}\right)\right]$$

This data was then imported into OptiGrating^®^ [26] to predict the reflected spectra. The effective refractive index and the central wavelength of the optical fibre used, SMF-28^®^, are shown in Table 4 [13].

**Figure 11:**
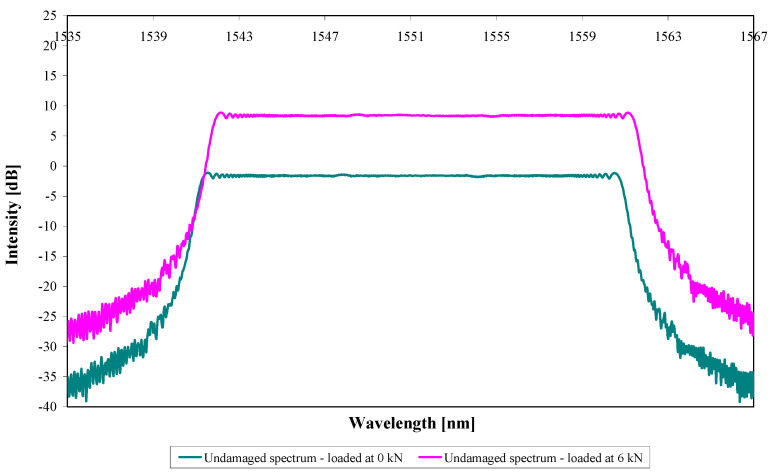
Predicted reflected spectra of the undamaged model when loaded at 0 kN and 6 kN.

Figure 11 shows the predicted reflected spectra corresponding to loads of 0 kN and 6 kN for the repaired specimen (before fatigue damage) using the strain distribution shown in Figure 10. The 6 kN reflected spectrum has been shifted +10 dB for easier visualisation relative to the 0 kN spectrum. Figure 11 shows a uniform reflected spectrum due to uniform strain along the position of the sensor (as shown in Figure 10) at 0 kN; for the 6 kN loading, the small undulations in the strain distribution are not sufficient to produce perturbations in the spectrum. The reflected spectrum is shifted 0.68 nm to higher wavelengths (an average of the measurements at the low- and high-wavelength ends, full-width at half-maximum) that correspond to a tensile strain of 680 µε (0.07%). However, the experimentally measured reflected spectra (before damage) at 6 kN show a slightly higher average tensile strain equivalent to 830 µε (0.08%); it is not entirely clear why there is a difference of this magnitude here, although the qualitative behaviour is correct.

### 3.2. Predicted Reflected Spectra of the Repaired Coupon Due to Fatigue Damage

The experimental results showed that the sensor could detect the growing fatigue crack when it was approximately 2 to 3 mm from the sensor position (Figure 7). From this point, a perturbation developed in the reflected spectra until the crack had grown about 2 mm beyond the sensor location, at that point the perturbation stabilised and a new perturbation at the high-wavelength end of the spectrum developed.

**Figure 12:**
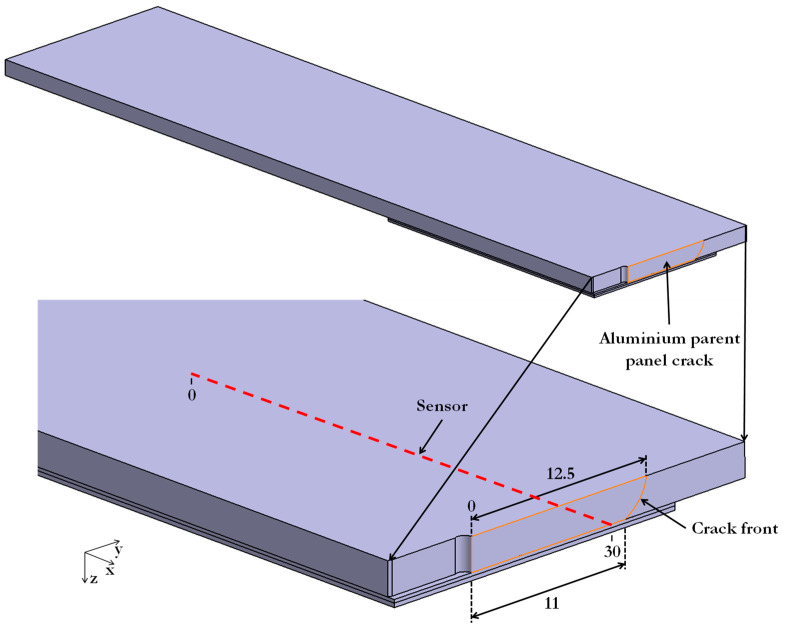
3D schematic of the damaged model showing a crack of 12.5 mm.

To model the stabilised spectra, a FE model was created with a crack that had grown beyond the location of the sensor by 2.5 mm (Figure 12). The crack length on the patched side was taken to be slightly smaller (11 mm). This was based on the work of Schubbe et al. [1] who performed a study on a similarly repaired system and tested tension-tension fatigue loading and found an elliptical crack front; the ratio of the crack lengths on the two aluminium surfaces was found by Schubbe et al. [1] to be 0.88, and this ratio was used here. Damage in the GFRP patch seen in Figure 4 was not considered because this was restricted to matrix cracking and possible fibre/matrix debonding, which would reduce the modulus of the composite material by about 20% at most [27]. The crack length of 11 mm on the patched face is 1 mm beyond the sensor, as shown in Figure 12. The crack front was created in the FE model by releasing the symmetry boundary condition of the nodes on the crack surface. The mesh for these models was kept similar to the undamaged models (Section 3.1) for consistency. However, the mesh refinement was extended from the notch tip to the crack tip for better accuracy.

**Figure 13:**
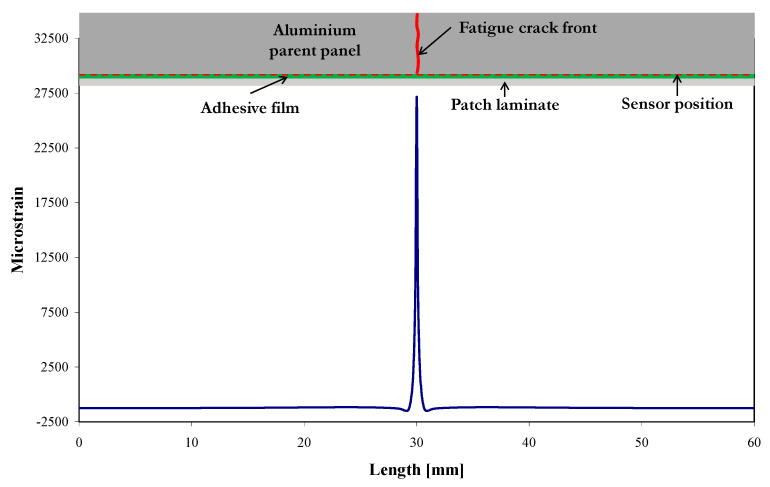
Predicted strain profile along the position of the 60 mm sensor in the damaged model (12.5 mm crack) when loaded at 6 kN.

Figure 13 shows the strain profile at the position of the sensor for the damaged model (i.e., having a crack of length 12.5 mm) loaded in tension to 6 kN. Here, it can be seen that at the position of the crack, there is a sharp increase of strain up to approximately 27,500 µε (2.8%). This is not surprising, since the crack has grown beyond the location of the sensor so that, in effect, the sensor and the patch are bridging the crack. The peak strain is probably due to a combination of a bi-material singularity and the stress concentration caused by local load transfer from the cracked aluminium to the patch. In the remaining parts of the sensor, the strain is uniform with a value of −1250 µε, which is of the same order of magnitude as the uniform strain of the 6 kN profile in Figure 10.

**Figure 14:**
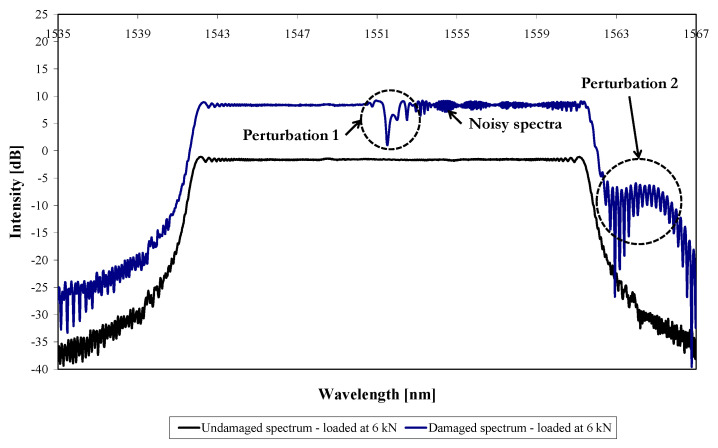
Predicted reflected spectra of the undamaged and the damaged models for a crack length of 12.5 mm loaded at 6 kN.

Figure 14 shows the predicted reflected spectra (obtained from Optigrating^®^) of a repaired coupon (undamaged) and for a repaired coupon with a fatigue crack (in the aluminium) of 12.5 mm, both loaded at 6 kN (the damaged specimen has been shifted by +10 dB on the intensity scale). Figure 14 shows that for a crack that has grown beyond the sensor location, perturbations very similar to that seen in the experimental results of Figure 7a,b are predicted. “Perturbation 1” is located at the centre of the reflected spectrum as in the experimental results. In addition, there is also noise in the spectrum extending from Perturbation 1 towards the high-wavelength end of the spectrum, again as in the experimental results. In addition to this, a second perturbation, labelled “Perturbation 2” is apparent at the high-wavelength end, again as seen in the experimental results.

**Figure 15:**
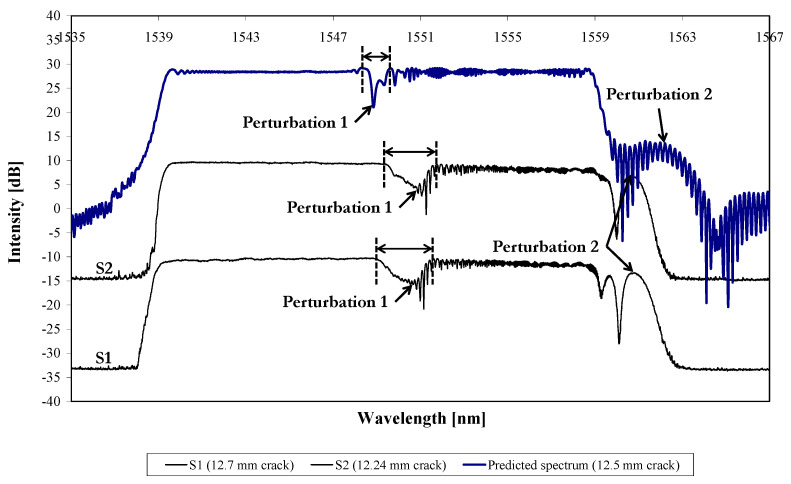
Recorded/predicted reflected spectra of the damaged specimens (S1 and S2) and damaged model loaded at 6 kN.

However, there are a few minor differences between the predicted and recorded spectrum, and Figure 15 shows the recorded and predicted reflected spectra for comparison. The spectra have again been shifted on the intensity axis for easier visualisation, and the predicted spectrum is aligned with the recorded spectra at the low-wavelength end, again to facilitate comparison. Figure 15 shows that the shape of “Perturbation 1” in the predicted spectrum is narrower than that for the two experimental results. In addition, the position of “Perturbation 1” within the predicted spectrum is at the centre of the spectrum, whereas the recorded perturbations are shifted slightly to higher wavelengths (measured at half-width of each perturbation). Finally, Figure 15 shows that the predicted “Perturbation 2” is different in shape to the experimentally recorded “Perturbation 2”. These differences probably relate to the idealisation of the strain field, which the FE model provides, ignoring experimental complications such as local variations in sensor position and the fatigue crack path. In addition, some of the differences may be due to the details of damage of the aluminium–adhesive interface in the vicinity of the crack.

It should perhaps be pointed out that it may be possible to detect the approach of a growing fatigue crack in a patch repair using a conventional FBG sensor. This would certainly be attractive on cost grounds since CFBGs are more expensive than FBGs. On the other hand, in connection with damage, it is generally much easier to interpret changes to CFBG spectra in comparison with the complexities of FBG spectra.

## 4. Concluding Remarks

Fatigue crack growth was observed in a GFRP patch-repaired aluminium coupon under tension-tension fatigue loading. Prior to repair, a central notch was introduced in the aluminium specimens to facilitate damage initiation. A pre-cured transparent patch, fabricated from a single ply of eight-harness satin fabric and epoxy resin, was bonded to one face of the specimen using an aerospace film adhesive. Chirped FBG sensors were embedded in the bond-line adjacent to the aluminium parent panel (i.e., within the bond-line, between the panel and the patch).

During fatigue loading, fatigue cracks developed in the aluminium panel from the notch tips and grew approximately perpendicular to the loading direction. Finite-element modelling of the undamaged and the damaged coupons was carried out using solid elements with a refined 3D mesh. For the damaged coupons, the model considered a crack that had just passed the position of the sensor. In terms of major features within the spectrum, a good agreement was found between the predicted spectra and the experimental spectra.

It can be concluded that, in terms of the experimental results, the reflected spectra from the embedded CFBG sensor clearly indicated the approach of a fatigue crack when the crack front was about 2 to 3 mm from the sensor position. In the early stages of crack growth, the sensor did not detect any strain field change and therefore the reflected spectra remained uniform. After the crack was within approximately 3 mm of the sensor, a perturbation was formed in the reflected spectra, which increased in size with further crack growth. After the crack had passed the sensor, the shape of the perturbation remained essentially constant. Consequently, it can be concluded that CFBG sensors are able to detect crack growth in a composite patch-repaired panel and to indicate when the crack has passed the location of the sensor by the stability of the main perturbation.

## Figures and Tables

**Table 1:** Glass fibre reinforced polymer (GFRP) laminate properties.

E_1_ = 21,000 MPa	ν_12_ = 0.183	G_12_ = 3700 MPa
E_2_ = 21,000 MPa	ν_13_ = 0.0305	G_13_ = 3500 MPa
E_3_ = 8550 MPa	ν_23_ = 0.075	G_23_ = 3500 MPa

**Table 2:** FM-73 OST and Al-2014 properties.

E_FM-73 OST_ = 2000 MPa	ν = 0.4
E_Al_ = 72,400 MPa	ν = 0.33

**Table 3:** Coefficient of thermal expansion (CTE) of the different materials considered.

Material	CTE [×10^−6^ °C^−1^]
Al-2014-T6	23 [21]
GFRP	8 ^1^ [22]
FM-73 OST	50 [23]

^1^ in the longitudinal direction.

**Table 4:** SMF-28^®^ optical properties.

Poisson’s ratio	0.16
Strain optic coefficients:	
*P*_11_	0.113
*P*_12_	0.252
Effective refractive index of CFBG sensor	1.449
Central wavelength (nm)	1550

## Data Availability

Not applicable.

## References

[1] Schubbe J.J., Mall S. (1999). Investigation of a cracked thick aluminum panel repaired with a bonded composite patch. Eng. Fract. Mech..

[2] Schubbe J.J., Mall S. (1999). Modeling of cracked thick metallic structure with bonded composite patch repair using three-layer technique. Compos. Struct..

[3] Soutis C., Duan D.M., Goutas P. (1999). Compressive behaviour of CFRP laminates repaired with adhesively bonded external patches. Compos. Struct..

[4] Soutis C., Hu F.Z. (1997). Design and performance of bonded patch repairs of composite structures. Proc. Inst. Mech. Eng. Part G J. Aerosp. Eng..

[5] Sun C.T., Klug J., Arendt C. (1996). Analysis of cracked aluminum plates repaired with bonded composite patches. AIAA J..

[6] Baker A., Gunnion A.J., Wang J., Chang P. (2016). Advances in the proof test for certification of bonded repairs—Increasing the Technology Readiness Level. Int. J. Adhes. Adhes..

[7] Yashiro S., Wada J., Sakaida Y. (2017). A monitoring technique for disbond area in carbon fiber–reinforced polymer bonded joints using embedded fiber Bragg grating sensors: Development and experimental validation. Struct. Health Monit..

[8] Kakei A., Epaarachchi J.A., Islam M., Leng J. (2018). Evaluation of delamination crack tip in woven fibre glass reinforced polymer composite using FBG sensor spectra and thermo-elastic response. Measurement.

[9] Rajabzadeh A., Heusdens R., Hendriks R., Groves R. (2019). Characterisation of transverse matrix cracks in composite materials using fibre Bragg grating sensors. J. Lightwave Technol..

[10] Chen J., Wang J., Li X., Sun L., Li S., Ding A. (2020). Monitoring of temperature and cure-induced strain gradient in laminated composite plate with FBG sensors. Compos. Struct..

[11] Palaniappan J., Wang H., Ogin S.L., Thorne A., Reed G.T., Tjin S.C. (2005). Use of conventional and chirped optical fibre Bragg gratings to detect matrix cracking damage in composite materials. J. Phys..

[12] Palaniappan J., Ogin S.L., Thorne A.M., Reed G.T., Crocombe A.D., Capell T.F., Tjin S.C., Mohanty L. (2008). Disbond growth detection in composite-composite single-lap joints using chirped FBG sensors. Compos. Sci. Technol..

[13] Okabe Y., Tsuji R., Takeda N. (2004). Application of chirped fiber Bragg grating sensors for identification of crack locations in composites. Compos. Part A Appl. Sci. Manuf..

[14] Palaniappan J., Wang H., Ogin S.L., Thorne A.M., Reed G.T., Crocombe A.D., Rech Y., Tjin S.C. (2007). Changes in the reflected spectra of embedded chirped fibre Bragg gratings used to monitor disbonding in bonded composite joints. Compos. Sci. Technol..

[15] Capell T.F., Palaniappan J., Ogin S.L., Thorne A.M., Reed G.T., Crocombe A.D., Tjin S.C., Wan Y., Guo Y. (2009). Detection of defects in as manufactured GFRP-GFRP and CFRP-CFRP composite bonded joints using chirped fibre Bragg grating sensors. Plast. Rubber Compos..

[16] Rito R.L., Crocombe A.D., Ogin S.L. (2017). Health monitoring of composite patch repairs using CFBG sensors: Experimental study and numerical modelling. Compos. Part A Appl. Sci. Manuf..

[17] Marsden W.M. (1996). Damage Accumulation in a Woven Fabric Composite. Ph.D. Thesis.

[18] Xu M.G., Archambault J.L., Reekie L., Dakin J.P. (1994). Discrimination between strain and temperature effects using dual-wavelength fibre grating sensors. Electron. Lett..

[19] Jones F.R., Mulheron M., Bailey J.E. (1983). Generation of thermal strains in GRP. J. Mater. Sci..

[20] Palaniappan J., Wang H., Ogin S.L., Thorne A., Reed G.T., Tjin S.C., McCartney L.N. (2006). Prediction of the reflected spectra from chirped Bragg gratings embedded within cracked crossply laminates. Meas. Sci. Technol..

[21] ASM “Al 2014-T6 Material Data Sheet”. http://asm.matweb.com/search/SpecificMaterial.asp?bassnum=MA2014T6.

[22] AASHTO LRFD (2009). Bridge Design Guide Specifications for Gfrp-Reinforced Concrete Bridge Decks and Traffic Raillings.

[23] Sabelkin V., Mall S., Hansen M.A., Vandawaker R.M., Derriso M. (2007). Investigation into cracked aluminium plate repaired with bonded composite patch. Compos. Struct..

[24] Jülich F., Roths J. Determination of the effective refractive index of various single mode fibres for fibre Bragg grating sensor applications. Proceedings of the SENSOR+TEST Conferences.

[25] Ouellette F. (1987). Dispersion cancellation using linearly chirped Bragg grating filters in optical waveguides. Opt. Lett..

[26] (2008). Optigrating.

[27] Rito R.L. (2015). Monitoring Damage Development in Composite Repairs Using Chirped Fibre Bragg Grating Sensors. Ph.D. Thesis.

